# Preserving centromere identity: right amounts of CENP-A at the right place and time

**DOI:** 10.1007/s10577-025-09780-4

**Published:** 2025-09-30

**Authors:** Zofia Pukało, Bethan Medina-Pritchard, Maria Alba Abad, A. Arockia Jeyaprakash

**Affiliations:** 1 School of Biological Sciences, University of Edinburgh, Edinburgh, EH9 3BF UK; 2 MRC Human Genetics Unit, Institute of Genetics and Cancer, University of Edinburgh, Edinburgh, UK; 3 Gene Center Munich, Ludwig-Maximilians-Universität München, Munich, 81377 Germany

**Keywords:** CENP-A, Centromere, Mis18, Transcription, Regulation, Ubiquitination

## Abstract

Four decades ago, the discovery of centromere protein-A (CENP-A) marked a pivotal breakthrough in chromosome biology, revealing the epigenetic foundation of centromere identity. CENP-A, a histone H3 variant, directs the formation of the microtubule-binding kinetochore complex, designating the chromosomal site for its assembly and underpins the accurate partitioning of genetic material during cell division. Errors in cell division can give rise to DNA instability and aneuploidy, implicated in human diseases such as cancer. Therefore, discovering the underlying pathways and mechanisms responsible for the formation, regulation and maintenance of the centromere is important to our understanding of genome stability, epigenetic inheritance, and in providing the knowledge to help generate possible treatments and therapeutics. Here, we review various molecular pathways and mechanisms implicated in maintaining centromere identity and highlight some of the key outstanding questions with a focus on the human centromere.

## Centromere: specialised chromatin with CENP-A as epigenetic mark

Centromeres are specialised chromosomal regions that act as a platform for the formation of the kinetochore, a large protein assembly capable of binding to spindle microtubules during mitosis (and meiosis). This physical attachment, monitored by the spindle assembly checkpoint (SAC), facilitates bipolar attachment and chromosome alignment at the metaphase plate (Musacchio & Salmon 2007). Upon chromosome biorientation, the silencing of the SAC triggers the onset of anaphase (indirectly via the anaphase promoting complex), upon which the chromosomes separate and move towards opposite poles of the cell (Rieder & Salmon 1998) and reviewed in (McAinsh & Kops 2023). Failure of the centromere and kinetochore to perform their functions properly can cause aneuploidy and genomic instability, which may contribute to congenital disorders and increase the risk of cancer (Bodor et al. 2014; Santaguida & Amon 2015; Shrestha et al. 2017). Due to its essential role in cell division, how centromeres differ from other regions of the chromosomes, and what features allow them to perform their function, have long been of interest.

**Fig. 1:**
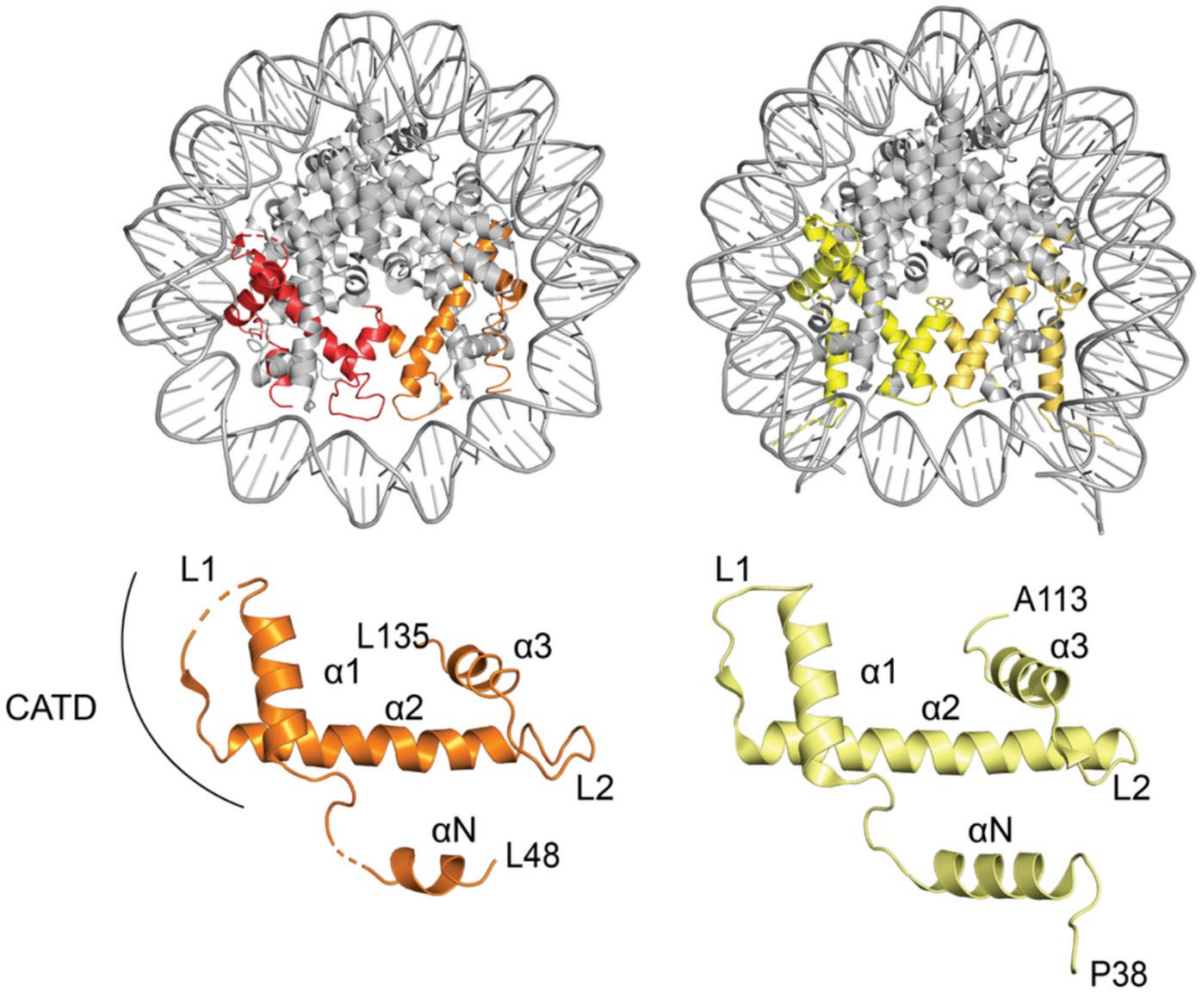
Crystal structures of CENP-A and H3 containing nucleosomes**.** Top panels display the nucleosome containing CENP-A (left, PDB: 3AN2 (Tachiwana et al. 2011a, b)) with CENP-A shown in red and orange, and the nucleosome containing H3 (right, PDB: 3AV2 (Tachiwana et al. 2011a, b)) with H3 shown in yellow and gold. Bottom panels display a single CENP-A or H3 molecule with the different secondary structure elements labelled

In the 1980s, work by Bill Earnshaw led to the identification of the first known centromeric proteins: centromere protein-A (CENP-A), CENP-B, and CENP-C. This laid the foundation for the discovery that the human centromere is not denoted by a specific DNA sequence, but rather epigenetically defined through an increase in the presence of nucleosomes containing a histone H3 (H3) variant, CENP-A (Earnshaw et al. 2013; Earnshaw & Rothfield 1985; Palmer et al. 1991, 1987), which is enriched 50 times at centromeres compared to other regions of the chromosome (Bodor et al. 2014). The discovery in the 1990s of neocentromeres, new centromeres that form in regions of the chromosomes that are not normally associated with centromeres, also strengthen the notion that centromeres are epigenetically defined (Barry et al. 1999; Voullaire et al. 1993; Wandall et al. 1998). It has since been shown that a vast number of organisms utilise CENP-A (or a CENP-A homologue) as an epigenetic way of defining their centromere, though there are some exceptions, such as *Trypanosomatida,* which do not have CENP-A (Lowell & Cross 2004). CENP-A is the most divergent variant of the histone H3’s, with less than ~ 50% conservation in humans compared to the canonical H3.1 (Palmer et al. 1991, 1987). CENP-A and H3 both have nearly identical secondary structure elements: an αN helix, followed by histone fold domain comprised of an α1 helix, a loop region (L1), α2 helix, a second loop (L2) and a final α3 helix (Fig. 1). However, the N- and C-terminal tails are highly divergent (Sharma et al. 2019), contributing to distinct post-translational modifications and protein interactions. Additionally, CENP-A has a shorter αN helix, and a stretch of amino acid sequence known as the CENP-A targeting domain (CATD) spanning loop L1 to the end of α2 helix of the histone fold domain (Fig. 1), which is required for localisation to the centromere (Black et al. 2007). Although the structures of canonical and CENP-A nucleosomes are very similar, some key differences include the position of the L1 loop, and the flexibility of DNA at the DNA entry-exit site of CENP-A nucleosomes (Sharma et al. 2019; Tachiwana et al. 2011a, b). These differences result in the CENP-A nucleosome being more unstable than its H3 counterpart (Conde e Silva et al. 2007). It has been proposed that the flexibility of the DNA in the CENP-A nucleosome is important for its function (Roulland et al. 2016).

### Centromere organisation

Organisation of the centromere varies greatly between different species. Unlike most organisms that contain centromeres at a single discrete site, holocentric organisms such as *C. elegans* deposit CENP-A containing nucleosomes in transcriptionally defined sites rather than specific centromeric loci (Gassmann et al. 2012; Senaratne et al. 2022). Monocentric centromeres can be further divided into point centromeres, such as found in *S. cerevisiae*, which use a defined DNA sequence to form the centromere (Furuyama & Biggins 2007), and regional centromeres which consist of much larger repetitive DNA sequences which facilitate, but are not sufficient on their own, for the formation of the centromere (reviewed in (Sundararajan & Straight 2022)).

Humans have regional centromeres consisting of megabases of DNA composed of arrays of AT-rich, ~ 171 bp alpha satellite DNA tandem repeats arranged in a head-to-tail orientation (also known as higher order repeats, HOR) onto which CENP-A nucleosomes are formed (reviewed in (Sundararajan & Straight 2022)). These are flanked by heterochromatic regions, known as pericentromeres, with lower-order repeats. Estimates suggest that the human centromere contains, on average ~ 200 CENP-A nucleosomes (accounting for ~ 4% of nucleosomes at the centromere) (Bodor et al. 2014). Roughly half of the alpha satellite DNA monomers contain a 17 bp sequence known as a CENP-B box, which acts as a binding site for CENP-B. CENP-B binds DNA utilising a domain containing two helix-turn-helix motifs and homodimerises through its C-terminal dimerisation domain (Cooke et al. 1990; Masumoto et al. 1989). Though CENP-B is thought to be non-essential for centromere formation, due to the observation that the Y chromosome and neocentromeres lack any CENP-B boxes, several studies have suggested that CENP-B contributes to de novo centromere assembly and centromere maintenance (Chardon et al. 2022; Fachinetti et al. 2015; Fujita et al. 2015; Nagpal et al. 2023; Okada et al. 2007). It has been proposed that this is due to CENP-B’s ability to bind nucleosomal CENP-A (Fujita et al. 2015; Okada et al. 2007) and CENP-C at the kinetochore (Fachinetti et al. 2015; Suzuki et al. 2004), resulting in the stabilisation of both proteins at the centromere. More recent work, which has taken advantage of capture-C and super-resolution imaging, has proposed a bipartite model for the centromere during mitosis. They observed that the centromere is split into 2 domains, which are stabilised by cohesion, that in turn act as a platform form the formation to a bipartite kinetochore. The bipartite kinetochore can bind to 2 discrete bundle of microtubules, which could have implications in our understanding on how microtubules attach to chromosomes and the process of error correction (Sacristan et al. 2024).

### Centromere: chromosomal locus that drives chromosome segregation via the kinetochore

**Fig. 2:**
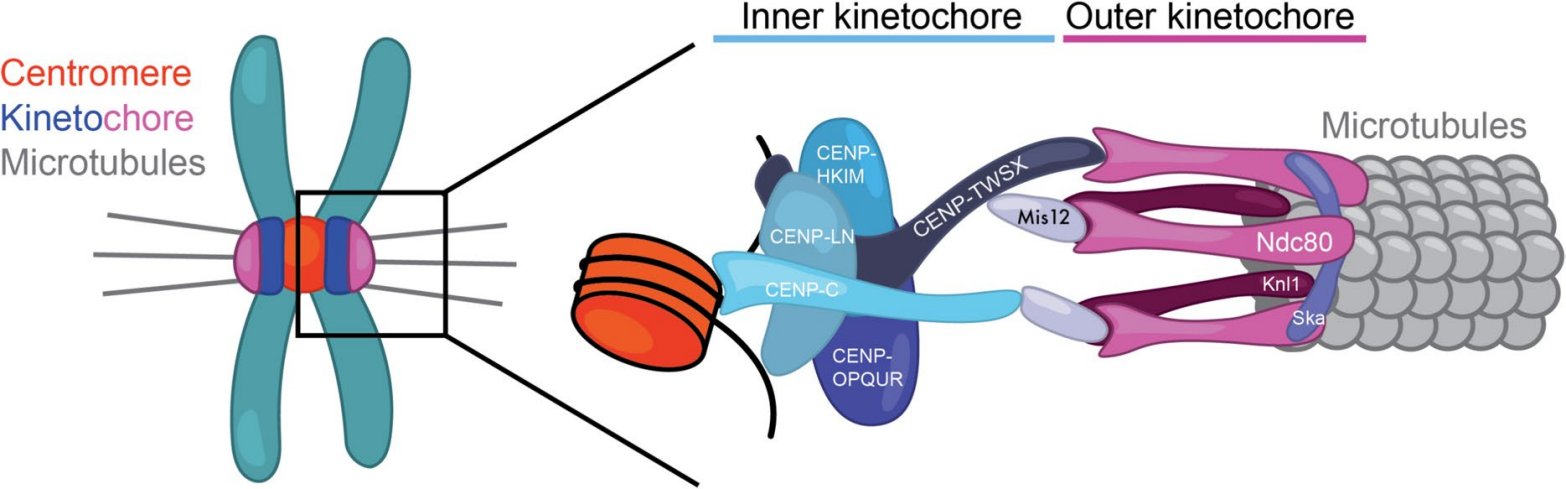
Organisation of the CCAN. The figure shows a schematic representation of the centromere, inner and outer kinetochore in humans. (Yatskevich et al. 2022)

CENP-A nucleosomes specify the chromosomal locations for the formation of the kinetochores, a multi-subunit protein assembly capable of binding up to 20 microtubules in Humans. The Human kinetochore is made up of the inner kinetochore, comprising the 16 subunits of the constitutive centromere associated network (CCAN); and the outer kinetochore, which contains the Knl1/Mis12/Ndc80 (KMN) network and the spindle and kinetochore-associated (Ska) complex (Fig. 2, reviewed in (Musacchio & Desai 2017)).

The CCAN assembles on the core region of the centromere and remains associated with it throughout the cell cycle, serving as a foundational platform for kinetochore assembly. The CCAN can be divided into different subcomplexes based on their structural organisation and ability to form stable sub-assemblies: CENP-C (Klare et al. 2015), CENP-O/P/Q/U/R (Hori et al. 2008a, b), CENP-L/N (McKinley et al. 2015), CENP-H/I/K/M (Weir et al. 2016), and CENP-T/W/S/X (Amano et al. 2009; Hori et al. 2008a, b). This arrangement likely allows the CCAN to be dynamic and undergo slight rearrangements in composition and conformation during the cell cycle. Of these components, CENP-C plays a pivotal role, as it can interact directly with CENP-A and serves as an anchor for various components of the CCAN (Mis12, CENP-T/W, and CENP-H/I/K/M complexes) (Ali-Ahmad et al. 2019; Ariyoshi et al. 2021; Carroll et al. 2010; Falk et al. 2015; Guse et al. 2012; Kato et al. 2013; Milks et al. 2009). CENP-N is also capable of directly interacting with CENP-A nucleosome, outside of the CCAN complex (Chittori et al. 2018). However, the timing and functional relevance of this interaction are yet to be determined. For an in-depth review of inner kinetochore structures of human and yeast, refer to (Yatskevich et al. 2023).

### Mechanisms of centromere maintenance: cell cycle-controlled CENP-A replenishment compensates replication-mediated CENP-A dilution

During DNA replication, the CENP-A nucleosomes are distributed between the old and newly replicated DNA, effectively reducing the amount of CENP-A nucleosomes at centromeres by half. Therefore, new CENP-A, which is mainly synthesised in G2, needs to be actively reloaded onto the centromere to restore the correct amount of CENP-A nucleosomes and preserve centromere identity (Dunleavy et al. 2011; Jansen et al. 2007). In humans, this deposition is uncoupled from DNA replication and occurs at the end of mitosis/early G1 (Jansen et al. 2007). As maintaining centromere identity is critical for the assembly of the kinetochore at the correct chromosomal locus, the following key conceptual questions have been on centre stage in the field for a long time: What are the molecular players and associated processes that achieve CENP-A deposition?; What are the licensing mechanisms responsible for restricting CENP-A deposition to a specific window of the cell cycle?; and What are the molecular mechanisms underlying the inheritance of centromere identity during DNA replication?

A vast body of work on yeast, fruit flies and humans have identified several critical players required for CENP-A deposition (Chen et al. 2014; Dunleavy et al. 2009; Foltz et al. 2009; Fujita et al. 2007; Hayashi et al. 2004; Mizuguchi et al. 2007; Phansalkar et al. 2012; Pidoux et al. 2009; Williams et al. 2009) reviewed in (Stellfox et al. 2013)). In humans, CENP-A deposition requires the missegregation protein 18 (Mis18) complex, an octameric complex consisting of Mis18α, Mis18β and Mis18 binding protein 1 (Mis18BP1) (Fujita et al. 2007), and the Holliday junction recognition protein (HJURP) chaperone (Dunleavy et al. 2009; Foltz et al. 2009). These proteins collectively constitute the CENP-A loading machinery.

**Fig. 3:**
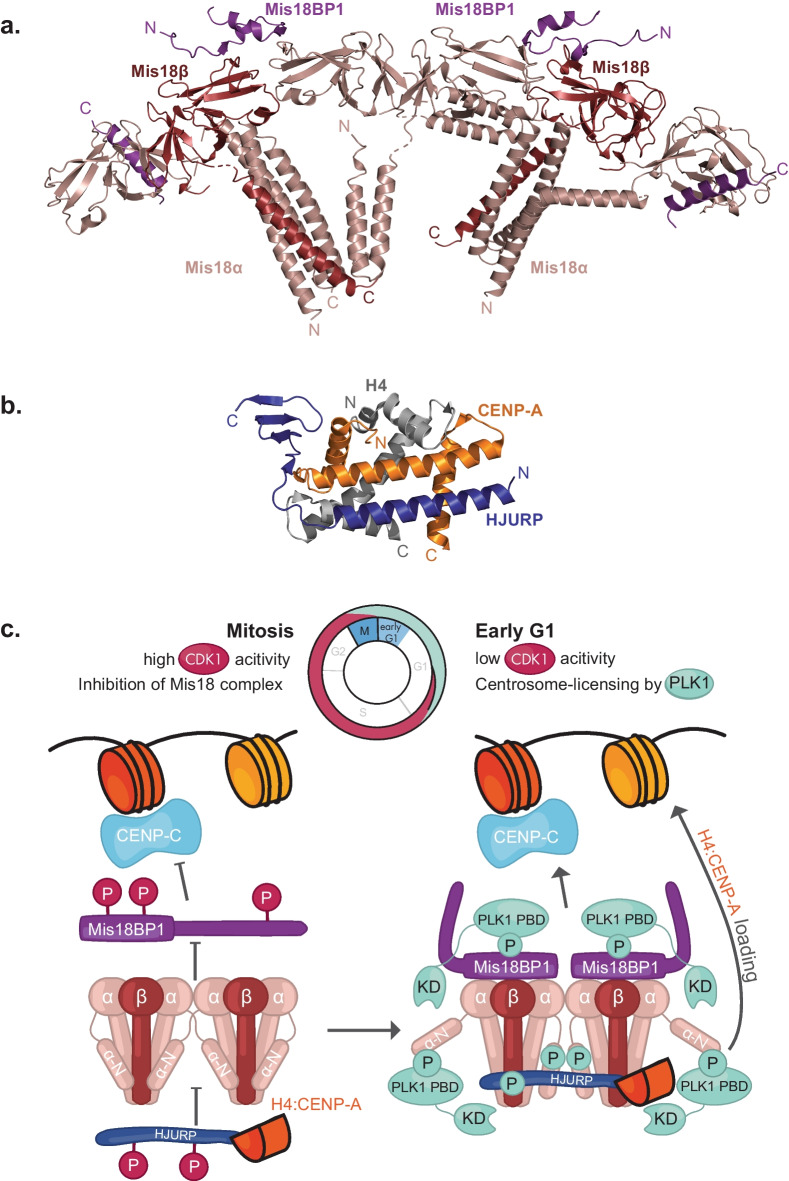
Mis18 and CENP-A deposition regulation. **a**) Model of Mis18 complex combining information from crystal structures, AlphaFold and crosslinking data (Mis18α in light pink, Mis18β in dark pink and Mis18BP1 in purple PDB: 9A8G (Thamkachy et al. 2023)). **b**) Crystal structure of the N-terminal region of HJURP (blue) interacting with CENP-A/H4 (orange and grey, respectively, PDB: 3R45 (Hu et al. 2011)). **c**) Mechanism of regulation of CENP-A deposition is established by phosphorylation of the loading machinery by CDK1 and PLK1 during the cell cycle (Conti et al. 2024; Parashara et al. 2024)

***Mis18 complex assembly and centromere association***: Both Mis18α and Mis18β contain a globular domain called the ‘YIPPEE’ domain followed by a C-terminal α-helix. In addition, Mis18α has an additional N-terminal α-helical region which is not present in Mis18β. The YIPPEE domain of Mis18α can form homodimers, whilst the YIPPEE domain of Mis18β can only form a heterodimer with Mis18α YIPPEE (Spiller et al. 2017; Subramanian et al. 2016). The C-terminal α-helices of Mis18α and Mis18β form a triple helical bundle consisting of two Mis18α and one Mis18β. Together, these oligomerisation regions form a hetero-hexamer of four Mis18α and two Mis18β (Pan et al. 2017; Spiller et al. 2017) (Fig. 3 A). The N-terminal helical region of Mis18α folds back and interacts with the triple helical bundle formed by the C-terminal α-helices of Mis18α and Mis18β (Conti et al. 2024; Parashara et al. 2024; Thamkachy et al. 2023). The Mis18αβ hexamer then forms an octameric complex by binding two Mis18BP1’s through the YIPPEE domains of Mis18αβ and the N-terminal region (spanning 130 amino acid residues) of Mis18BP1 (Pan et al. 2017; Spiller et al. 2017) (Fig. 3A). The Mis18 complex interacts with HJURP through the C-terminal triple helical bundle of Mis18αβ (Pan et al. 2019; Walstein et al. 2025). HJURP chaperones CENP-A/H4 dimer to the centromere (via the Mis18 complex) by binding to the CATD of CENP-A via its N-terminal CENP-A binding domain (CBD) (Hu et al. 2011) (Fig. 3B). However, the mechanism by which the CENP-A loading machinery is targeted to the centromere remains largely unclear. Specific components of the CCAN, CENP-C and CENP-I, both critical for CCAN stability, have been implicated in the centromere recruitment of the Mis18 complex (Dambacher et al. 2012; French & Straight 2019; McKinley & Cheeseman 2014; Mitra et al. 2020; Moree et al. 2011; Shono et al. 2015). Direct interaction between CENP-C and HJURP has also been suggested to contribute to the centromere recruitment of the CENP-A loading machinery (Flores Servin et al. 2023; French et al. 2017; Tachiwana et al. 2015). A recent preprint from Walstein et al., has identified several interaction footprints (including the SANTA domain) on Mis18BP1 that likely engage with multiple kinetochore-associated binding determinants (CENP-C, CENP-HIKM, and yet to be identified additional determinants) upon Mis18BP1 dimerisation (Walstein et al. 2025).

***Temporal regulation of the Mis18 complex assembly and CENP-A deposition***: The process of CENP-A deposition first requires the formation of the Mis18 complex at the correct time and place, which is regulated by the kinase CDKs and Polo-like kinase 1 (PLK1) (Conti et al. 2024; French & Straight 2019; McKinley & Cheeseman 2014; Pan et al. 2017; Parashara et al. 2024; Silva et al. 2012; Spiller et al. 2017; Stankovic et al. 2017) (Fig. 3C). Phosphorylation of Mis18BP1 at residue T40 and S110 by CDK prevents its interaction with Mis18αβ until the end of mitosis, when CDK activity drops. This regulation prevents premature loading of CENP-A outside of late mitosis/G1 (Pan et al. 2017; Spiller et al. 2017). Phosphorylation of Mis18BP1 at T653 inhibits its centromeric localisation (Stankovic et al. 2017; Walstein et al. 2025). CDK phosphorylation at S210/S211 and S412 of HJURP also negatively regulates its localisation to the centromere (Stankovic et al. 2017).

PLK1 acts as a positive regulator of the CENP-A loading machinery and was proposed to license CENP-A loading by promoting the centromere localisation of the Mis18 complex during G1, mainly via the phosphorylation of Mis18BP1 (McKinley & Cheeseman 2014). However, the mechanistic understanding of PLK1-mediated licensing was provided only recently (Conti et al. 2024; Parashara et al. 2024). According to these recent studies, PLK1 associates with Mis18BP1 by directly recognising self-primed phosphorylation sites on Mis18BP1 at residues T78 and S93 via its Polo-Box domain, resulting in the centromere recruitment of PLK1 at G1. This then gives PLK1 access to the Mis18αβ, enabling it to phosphorylate and bind S54 located within the N-terminal α-helical region of Mis18α. When unphosphorylated, this region of Mis18α occludes the HJURP binding region of the Mis18αβ through intramolecular interactions (Conti et al. 2024; Parashara et al. 2024; Thamkachy et al. 2023), which is consistent with the observation that removal of the N-terminal region of Mis18α promotes HJURP binding (Pan et al. 2019). The PLK1 phosphorylation of Mis18α S54 triggers a conformational change that relieves this inhibitory conformation, making the C-terminal triple helical bundle of Mis18αβ more accessible for HJURP binding. Thus, it provides a mechanistic basis for PLK1-mediated conformational activation of the Mis18 complex as a licensing mechanism for CENP-A loading in G1 (Conti et al. 2024; Parashara et al. 2024).

However, there are still several open questions on processes beyond the centromere recruitment of the CENP-A loading machinery and mechanisms ensuring the replenishment of CENP-A to the original amount during each cell cycle. For example, how centromeric Histone H3.3, which is suggested to act as a ‘placeholder’ for CENP-A (Dunleavy et al. 2011; Shukla et al. 2018), is recognised and evicted to facilitate CENP-A loading and how the centromere-targeted CENP-A/H4 heterodimer is incorporated into the centromeric chromatin are some of the key open questions. Several proteins with diverse activities, such as Cdc42 (a small GTPase), MgcRacGAP, RSF (chromatin remodeller), and Ect2 have been implicated in stabilising CENP-A nucleosomes as part of a not well-understood ‘centromere maturation’ process (reviewed in (Mahlke & Nechemia-Arbely 2020)). Furthermore, mechanisms controlling the amount of CENP-A deposited at the centromere and mechanisms restricting centromere spreading are still poorly understood. It was proposed that HJURP-binding alters the oligomeric structure of the Mis18 complex and, as a consequence, the Mis18 complex dissociates from centromeres, limiting CENP-A deposition once per cell cycle (Nardi et al. 2016). Although attractive, this model was questioned as a subsequent in vitro study did not observe such a change in the oligomeric structure of the Mis18 complex upon HJURP binding (Pan et al. 2019). One of the alternative models is that the placeholder nucleosomes containing Histone H3.3 could act as a limiting factor for CENP-A disposition (Dunleavy et al. 2011; Shukla et al. 2018). Other factors that have been proposed to influence the level of CENP-A loading are the tension between the chromatids (Brown & Xu 2009; Mellone & Allshire 2003; Stankovic & Jansen 2017), and the regulation of the stability of CENP-A nucleosomes (van den Berg & Jansen 2023), which may allow for fine-tuning of CENP-A levels (further discussion on ubiquitination and sumoylation mediated control of CENP-A level is below). A recent preprint has suggested that the boundaries of the centromere are defined by heterochromatin containing H3K9me3, which may help maintain CENP-A levels and localisation in the centromere (Carty et al. 2025).

While the essentiality of maintaining centromere identity is conserved, the mechanistic bases of CENP-A loading and the players involved vary in different organisms. In *S. pombe* CENP-A (Cnp1) deposition happens in G2 (Lando et al. 2012), where the Spindle Pole Body (SPB, to *pombe* equivalent of the centrosome) plays an important role in CENP-A^Cnp1^ loading. Centromeres cluster at the nuclear periphery adjacent to the SPB (Funabiki et al. 1993), where other factors required for loading are also concentrated. The Mis18 complex in *pombe* consists of Mis18 (the only homologue of Mis18αβ), Mis16 (a homologue of RbAp46/48), Eic1/Mis19, and Eic2/Mis20 (suggested functional homologues of Mis18BP1) (Hayashi et al. 2014, 2004; Subramanian et al. 2014). Mis18 in *pombe* contains a YIPPEE domain, capable of dimerising, which is nearly identical to human Mis18 proteins, followed by a C-terminal α-helix, which can form a tetramer. However, it lacks the additional N-terminal regions found in human proteins (Subramanian et al. 2016). CENP-A^Cnp1^ is chaperoned to the centromere by the HJURP homologue Scm3 before deposition (Pidoux et al. 2009). Although Mis18 can bind with CENP-C (Cnp3), this interaction is not essential for CENP-A^Cnp1^ loading (Zhang et al. 2020). Mis18 can also interact with the N-terminus of Sad1, which brings it to the SPB, and is needed for maintenance of CENP-A^Cnp1^ at centromeres (London et al. 2023).

**Fig. 4:**
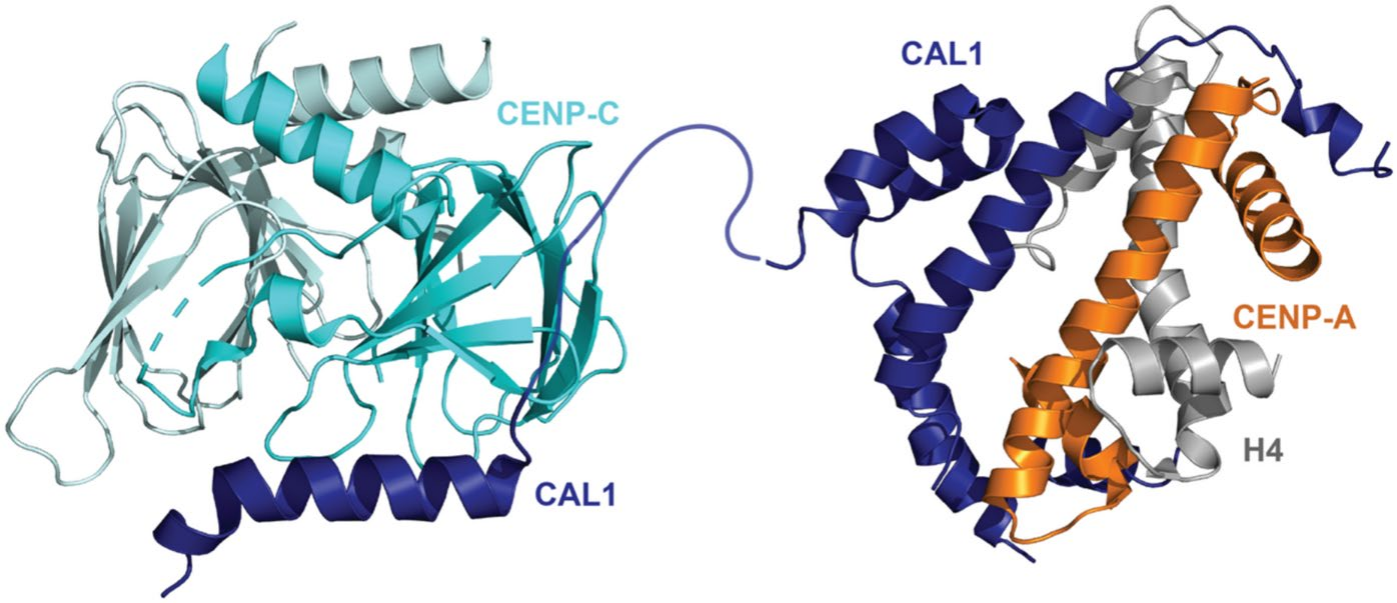
CAL1 interaction with CENP-C and CENP-A. Crystal structures showing CAL1 (dark blue) C-terminal interaction with CENP-C (light blue, PDB: 6XWV), and the CAL1 N-terminal interaction with CENP-A.^CID^/H4 (orange and grey, respectively, PDB: 6XWS) (Medina-Pritchard et al. 2020)

In *Drosophila* deposition of its CENP-A homologue (centromere identifier, CID) occurs between mitosis to G1 (Dunleavy et al. 2012; Hemmerich et al. 2008; Mellone et al. 2011). However, it does not possess any discernible homologues of HJURP or the Mis18 complex. Instead, CID is loaded by the chromosome alignment defect 1 (CAL1) protein (Chen et al. 2014; Phansalkar et al. 2012), which can perform the roles of the Mis18 complex and HJURP by binding CENP-A^CID^/H4 and CENP-C (Chen et al. 2014; Medina-Pritchard et al. 2020; Schittenhelm et al. 2010) (Fig. 4). Structural studies revealed that despite having little conservation at the amino acid level, the N-terminal of CAL1 interacts with CENP-A^CID^/H4 in a similar manner to HJURP, with some adaptive differences to account for the variation of the CENP-A^CID^ amino acid sequence (Medina-Pritchard et al. 2020). The C-terminus of CAL1 uses electrostatic and hydrophobic interactions to bind to a dimer of CENP-C via its Cupin domain, being capable of binding both CENP-C and CENP-A^CID^/H4 at the same time (Medina-Pritchard et al. 2020).

### Mechanisms of centromere maintenance during DNA-replication

Cellular processes influencing chromatin compaction and stability, such as DNA replication, pose a threat to centromere identity. This is due to the fact that during DNA replication the nucleosomes need to be briefly disassembled to allow the replication machinery/DNA polymerase to pass through, before being quickly reassembled onto either the leading or lagging DNA strands, resulting in gaps that need to be filled with new nucleosomes via de novo nucleosome assembly (reviewed in (Kulaeva et al. 2013)). This leads to an important question – how are the centromeric CENP-As of the parental strand retained and redistributed between the old and newly synthesised DNA strands? Two independent breakthrough studies showed that the replication machinery cooperates with the components of the de novo CENP-A loading machinery, such as HJURP, to achieve this (Nechemia-Arbely et al. 2019; Zasadzinska et al. 2018)). Particularly, the MCM2, a subunit of the DNA helicase MCM2-7 complex, can form a complex with HJURP, resulting in recognition of R63-K64 amino acids of CENP-A common to all types of H3 by MCM2, and the CATD of CENP-A by HJURP (Huang et al. 2015; Zasadzinska et al. 2018). This dual recognition of CENP-A during S-phase is important for the stable inheritance of CENP-A nucleosomes during DNA replication (Zasadzinska et al. 2018). The CCAN has also been proposed to have a critical role in retaining centromeric CENP-A, as CENP-C deletion in S-phase affected CENP-A levels at the centromere (Mitra et al. 2020; Nechemia-Arbely et al. 2019). However, if and how other members of the CENP-A loading machinery, such as the Mis18 complex or its subunits and/or chromatin remodellers, contribute to this process are still important open questions. Interestingly, the retention of CENP-A, mediated through replication, is restricted to the centromere, leading to a compelling proposal that replication serves as an error-correction mechanism to maintain centromere identity (Nechemia-Arbely et al. 2019). This is crucial as although centromeres are defined by an increase in the concentration of CENP-A nucleosomes, a large amount of CENP-A resides outside of the centromere, which could result in the formation of ectopic centromeres if non-centromeric CENP-A levels surpass a certain threshold (Bodor et al. 2014; Nechemia-Arbely et al. 2019).

## Novel players in centromere maintenance: centromeric transcription and DNA-damage

Like DNA replication, transcription and DNA damage also impose significant risks to centromere identity by perturbing local chromatin structure and nucleosome organisation, which may result in the potential loss of CENP-A nucleosomes. Emerging data have begun to provide insights into the molecular players counteracting this risk (such as FACT and Spt6 during transcription (Bobkov et al. 2020; Chen et al. 2015; Okada et al. 2009)), while suggesting possible direct roles of these processes in de novo CENP-A deposition itself.

### Centromeric transcription

It has been known since the late 70s that centromeres contain RNA (Heidemann et al. 1977; Rieder 1979), although it was not until the early 2000s that several studies showed that both centromeres and the flanking pericentromeric regions are transcribed by RNA polymerase II (RNA Pol-II; Chan et al. 2012; Liu et al. 2015), and reviewed (Perea-Resa & Blower 2018; Smurova & De Wulf 2018). Centromeric transcripts are now known to associate with centromeric chromatin and contribute to the regulation of centromere maintenance and function (Fukagawa & Earnshaw 2014; Talbert & Henikoff 2018; Zhu et al. 2023). Interestingly, in different organisms, the peak of expression at centromeres is observed at different phases of the cell cycle. In budding yeast, centromeric transcription occurs mainly during S phase (Ling & Yuen 2019). In mice, it peaks in G2/M (Ferri et al. 2009) while in human cells, it peaks during mitosis (Chan et al. 2012). It is believed that during the transcription process, centromeric transcription may play a role in promoting stable CENP-A incorporation by facilitating chromatin reorganisation (Quénet & Dalal 2014), while centromere RNA (cenRNA) transcripts could help target key centromeric proteins (Blower 2016; Ferri et al. 2009; Ling & Yuen 2019; McNulty et al. 2017; Quénet & Dalal 2014). The significance of centromeric transcription is not well understood due to the challenges associated with discovering the bona fide RNA–protein interactions. Many studies rely on indirect methods such as EMSA or RNA–protein co-pulldowns which are known to enrich for non-specific interactions as reviewed in (Cozzolino et al. 2021). Thus, potential interactions need to be revised carefully.

**Fig. 5:**
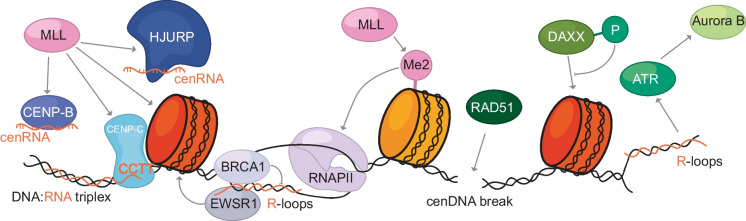
Role of transcription and DNA damage-related proteins on CENP-A loading and maintenance. MLL methyltransferases promote transcription at the centromere by RNA Pol-II by di-methylating H3 at Lys 4 (H3K4me2). This also affects the levels and recruitment of CENP-B, CENP-C, HJURP and CENP-A. RNA Pol-II also generates R-loops which have been shown to be important for CENP-A localisation and to recruit ATR kinase, which affects Aurora B activity and, hence, kinetochore formation. The DNA damage response pathway has also been linked through RAD51 to CENP-A deposition and maintenance

Using synthetic human artificial chromosomes (HACs), the Earnshaw lab demonstrated in 2016 that H3K4me2 is crucial for promoting centromeric transcription, and that loss of H3K4me2 leads to rapid loss of transcription linked with impaired CENP-A deposition and kinetochore instability (Bergmann et al. 2011; Malik et al. 2023; Molina et al. 2016). More recently, work from elsewhere has delved further into the mechanistic basis and found that MLL methyltransferases are responsible for depositing the H3K4me2 mark and play a crucial role in sustaining the unique epigenetic chromatin state of human centromeres (Malik et al. 2023). The same study shows that CRISPR-mediated downregulation of MLL methyltransferases causes a reduction in the centromeric levels of CENP-B and CENP-C, and also affects the centromeric recruitment of HJURP and CENP-A during early G1 (Malik et al. 2023) (Fig. 5).

CenRNAs have been suggested to interact with CENP-A, CENP-C and HJURP, and to recruit these proteins to the centromeres (McNulty et al. 2017; Quénet & Dalal 2014). CENP-C targeting transcript (CCTT), a non-centromere-derived long non-coding RNA (lncRNA), has been shown to interact with CENP-C and centromeric DNA, possibly through the formation of an RNA–DNA triplex (Zhang et al. 2022). So far, no sequence specificity has been identified as required for cenRNA to bind centromeric proteins.

RNAs could act as a guide to target proteins to centromeres. R-loops are three-stranded nucleic acid transcriptional by-products composed of a DNA:RNA hybrid and a displaced single-stranded DNA (Allison & Wang 2019; Santos-Pereira & Aguilera 2015) that are enriched at the promoter regions of actively transcribed genes, peaking at RNA-pol II start and termination sites. R-loops have important regulatory functions and are involved in gene expression, DNA repair and genome stability (reviewed in (Crossley et al. 2019)). Centromeric R-loops have been shown to be important for the recruitment of the ataxia telangiectasia mutated and Rad3-related (ATR) kinase, previously known for its role in DNA damage and replication stress response, to mitotic chromosomes. This novel R-loop-dependent ATR pathway activates ATR at centromeres, leading to Aurora B activation, and is thought to be important for kinetochore assembly and accurate chromosome segregation (Aze et al. 2016; Kabeche et al. 2018). Furthermore, a study by Kitagawa et al. showed that CENP-A localisation at the centromere is dependent on R-loop formation (Kitagawa et al. 2023). During interphase, the RNA-binding protein Ewing sarcoma breakpoint region 1 (EWSR1) tethers CENP-A to the centromere through its direct association with R-loops (Kitagawa et al. 2023). Yet another study revealed that the interaction of the tumour suppressor BRCA1 with centromeric chromatin is also R-loop dependent, which counteracts the accumulation of R-loops at centromeres (Racca et al. 2021). Cells lacking BRCA1 show impaired CENP-A localisation, higher levels of cenRNA transcription, increased centromeric breaks and Rad52-dependent hyper-recombination between alpha satellite repeats, all of which are linked to centromere instability and are dependent on R-loop formation (Racca et al. 2021) (Fig. 5). However, in numerous studies on R-loops, the S9.6 antibody is frequently employed to detect RNA:DNA hybrids (Boguslawski et al. 1986). This antibody has been shown to bind double-stranded RNA with similar affinity, and its immunofluorescent signal primarily originates from ribosomal RNA in fixed human cells (Hartono et al. 2018; Phillips et al. 2013). Therefore, it is crucial to validate the significance of R-loops in centromere maintenance using additional methods.

Overall, although the mechanistic basis remains to be determined, evidence suggests that RNA could be an essential part of centromere structure and function. However, we know little about cenRNA modifications and their function. A recent study has shown that CENP-A is a reader of m^6^A methylated cenRNA, a methylation enriched in cenRNA from cancer cell lines (Kang et al. 2024), and this modification contributes to maintaining CENP-A at centromeres during S-phase and to ensure centromere integrity in cancer cells. It is not yet clear whether the m^6^A modification plays a role in maintaining centromere integrity in normal cells. Future studies will shed light on the implications of cenRNA modifications and epitranscriptomic regulation in centromere maintenance and function.

### DNA damage-related proteins

Although essential for accurate chromosome segregation and crucial for cellular and organismal viability, centromeres are rapidly evolving, with alpha satellite sequences diverging even between closely related species (Balzano & Giunta 2020; Talbert et al. 2018). It is uncertain why such significant regions as centromeres would be prone to recombination as well as rapid change. The mechanisms behind this centromere paradox (Henikoff et al. 2001) are also still unknown.

Due to the highly repetitive nature of centromeric DNA, centromeres are believed to be fragile sites with high levels of intrinsic DNA breaks that have been associated with genome instability (Saayman et al. 2023; Scelfo et al. 2024), and reviewed in (Barra & Fachinetti 2018). The molecular mechanisms behind centromeric instability have recently become a significant area of research, gaining traction over the past decade (Nassar et al. 2023). The exact processes that cause mitotic errors resulting in structural chromosome changes are still not well understood. The fragility of the centromere is even more pronounced during DNA replication and replication stress, when incomplete or improper DNA replication can cause DNA breaks. Remarkably, centromeres are highly enriched in DNA-damage related proteins (Aze et al. 2016), and reviewed in (Nassar et al. 2023). The DNA recombinase RAD51 is a key component of the homologous recombination repair pathway, contributing to maintaining genome integrity (Stok et al. 2021; West 2003). Saayman and colleagues demonstrated that centromeric DNA breaks are induced both during active cell proliferation and during quiescence (Saayman et al. 2023). In quiescent cells, RAD51 resolves centromeric DNA breaks through its strand-exchange activity and has been suggested to regulate CENP-A deposition and centromere maintenance. The authors propose a model in which DNA breaks and the subsequent RAD51-dependent recombination maintain centromere specification through CENP-A deposition, suggesting that DNA damage alone may be sufficient to promote CENP-A deposition while possibly compromising genome stability (Saayman et al. 2023).

The ATR kinase, a key regulator of the DNA damage response, is also implicated in protecting CENP-A occupancy at interphase centromeres by regulating the phosphorylation of DAXX, the H3.3 chaperone (Trier et al. 2023). Reduction of ATR levels during interphase leads to CENP-A eviction, an aberrant increase in H3.3 at centromeres, defects in kinetochore formation and chromosome missegregation during mitosis (Trier et al. 2023). These results indicate that ATR kinase contributes to preserving centromere identity and genomic stability, even in the absence of DNA damage.

## Post-translational modifications as regulators of CENP-A maintenance

Since the CENP-A turnover on centromeres is an essential process for cell survival, it must be tightly regulated. Many studies have shown the importance of several histone phosphorylations and acetylations in CENP-A maintenance (Bailey et al. 2013; Conti et al. 2024; Hu et al. 2011; Parashara et al. 2024; Takada et al. 2017; Zeitlin et al. 2001), as reviewed in (Bailey et al. 2016; Fukagawa 2017; Srivastava et al. 2018). The post-translational modifications (PTMs) of CENP-A were shown, among others, to facilitate histone assembly onto nucleosomes (Almouzni & Cedar 2016; Hammond et al. 2017) and limit CENP-A deposition at the centromeres in the cell cycle dependent manner (Hu et al. 2011; Yu et al. 2015). However, the roles of other modifications, such as ubiquitination and SUMOylation, are less well understood. It is unclear how they contribute to centromere maintenance—they might directly influence the CENP-A amounts at centromeres by regulating the global CENP-A level or indirectly by altering protein interactions involved in maintaining centromere identity.

### Ubiquitin-mediated degradation of CENP-A

Ubiquitination is a PTM where a three-step enzymatic cascade that regulates a wide range of protein functions as reviewed in (Damgaard 2021) and (Zheng & Shabek 2017). Typically, the attachment of ubiquitin molecules to target proteins marks them for degradation by the proteasome (Jackson & Durocher 2013).

**Fig. 6:**
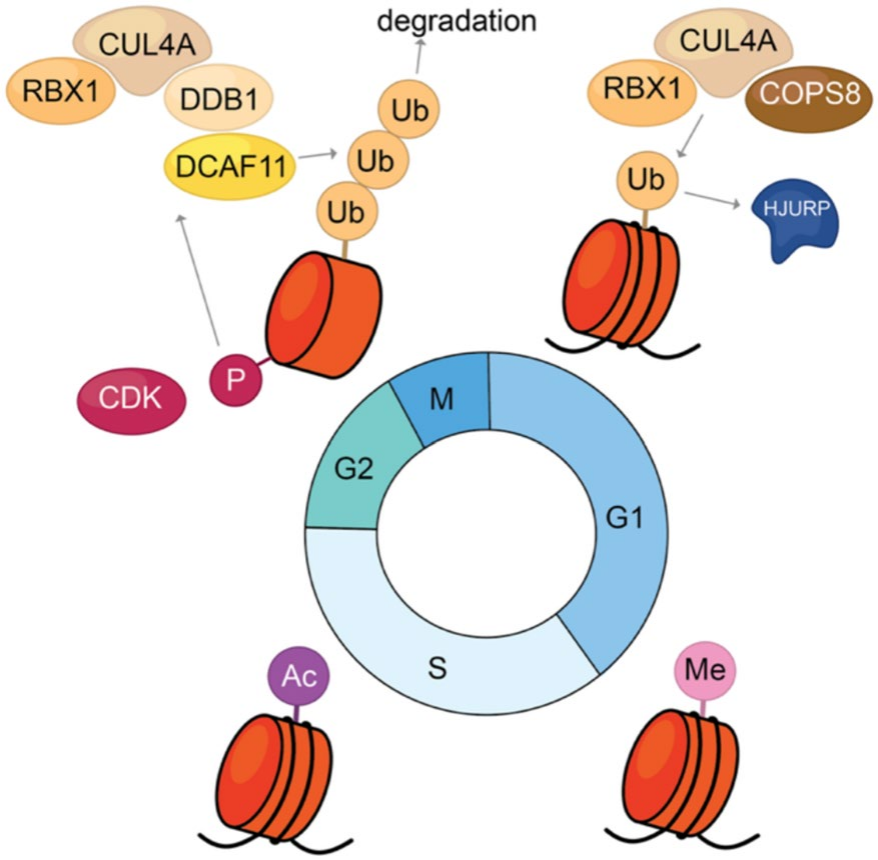
CENP-A modifications during the cell cycle. K124 in CENP-A can undergo different types of PTM during the cell cycle, including ubiquitination. The CUL4-DDB1-DCAF11 E3 causes poly-ubiquitination which leads to protein degradation by the proteasome. Whilst mono-ubiquitination performed by the CUL4A-RBX1-COPS8 E3 ligase promotes HJURP binding and is needed for CENP-A loading and maintenance

The regulation of CENP-A levels during cell cycle is essential as the overexpression of CENP-A leads to its mislocalisation at non-centromeric sites in yeast, flies and humans (Heun et al. 2006; Hildebrand & Biggins 2016; Shrestha et al. 2021). Overabundance of CENP-A ultimately results in genome instability, which is a hallmark of cancer (Athwal et al. 2015; Montes de Oca et al. 2015; Renaud-Pageot et al. 2022; Shrestha et al. 2021; Sullivan et al. 2011; Tomonaga et al. 2003). Hence, the better understanding of how CENP-A levels are balanced, for instance, through protein degradation, is of great interest. In yeast, multiple E3 ubiquitin ligases, including Psh1, Slx5, Ubr1, and SCF-Rcy, independently mediate the ubiquitin-dependent proteolysis of the CENP-A homolog Cse4 (Au et al. 2013; Cheng et al. 2017, 2016; Collins et al. 2004; Hewawasam et al. 2010; Ohkuni et al. 2016; Ranjitkar et al. 2010). Each ligase acts through distinct pathways to prevent Cse4 mislocalisation and ensure accurate chromosome segregation. Loss of these ligases leads to abnormal cell cycle progression, with Psh1 deletion causing the most severe defects (Cheng et al. 2017). In *Drosophila*, the SCF complex subunit Ppa (F-box protein) acts as an E3 ligase, promoting degradation of the CENP-A homolog CID (Moreno-Moreno et al. 2011). Phosphorylation of CENP-A^CID^ at S20 initiates its ubiquitination and subsequent degradation, restricting CENP-A^CID^ to centromeres and preventing its accumulation at non-centromeric sites (Huang et al. 2019). In humans, the CUL4-DDB1-DCAF11 (WDR23) E3 ligase complex mediates polyubiquitination of CENP-A, targeting it for degradation (Fig. 6). Phosphorylation of CENP-A at S68 by Cyclin B-CDK1 during G2/early M phase primes polyubiquitination at K49 and K124, facilitating proteasomal degradation and preventing ectopic CENP-A localisation during mitosis (Wang et al. 2021).

### Ubiquitination and centromere maintenance

In yeast and flies, E3 ligases such as Psh1 and Ppa recognise the CATD domain of CENP-A, which overlaps with the binding site of the CENP-A chaperone (Moreno-Moreno et al. 2011; Ranjitkar et al. 2010) (HJURP or its homologs, e.g., Scm3 in yeast (Camahort et al. 2007), CAL1 in flies (Chen et al. 2014)). Ubiquitination at this domain can inhibit chaperone binding, thereby regulating CENP-A incorporation into centromeres and targeting excess CENP-A for degradation (Cheng et al. 2017). However, in *Drosophila*, CUL3/RDX-mediated monoubiquitination of CENP-A^CID^, promoted by CAL1, stabilises CENP-A^CID^-CAL1 interaction facilitating CENP-A^CID^ loading onto centromeres. Inhibition of this ubiquitination disrupted centromere assembly and led to chromosome missegregation (Bade et al. 2014).

In humans, CENP-A K124 is also subjected to monoubiquitination by the CUL4A-RBX1-COPS8 E3 ligase (Niikura et al. 2015). This modification is suggested to be important for CENP-A deposition at centromeres, as monoubiquitin fusion can rescue centromere localisation defects caused by the K124R mutation (Niikura et al. 2019) This “signalling” ubiquitination is proposed to be required for proper interaction with the HJURP chaperone and centromere maintenance, rather than for degradation.

Curiously, K124 residue is also documented to be methylated and acetylated during the cell cycle (Bui et al. 2017; Srivastava & Foltz 2018). The acetylation happens at G1/S phase and regulates CENP-C interactions with CENP-A, mitotic integrity, and centromere replication timing (Bui et al. 2012). The acetyl group tightens the histone core which obstructs the C-terminus of CENP-A and inhibits CENP-C binding (Bui et al. 2017). During S phase, to ensure successful centromeric replication to progress correctly, the acetylation is exchanged for mono-methylation (Bui et al. 2017). The exact mechanism behind the K124-regulatory interplay between E3 ligases, acetyl-, and methyltransferases is unknown (Srivastava & Foltz 2018).

Outside of direct modifications of CENP-A, ubiquitination could also regulate other CCAN complex proteins that are heavily involved with CENP-A turnover. In human cells so far, only Mis18β has been shown to be regulated by ubiquitination. SCFβTrCP complex marks Mis18β for degradation in interphase. Unregulated levels of Mis18 complex proteins may lead to mislocalisation of CENP-A and aberrant segregation of proteins (Kim et al. 2014; Sethi et al. 2024).

### The role of SUMOylation in CENP-A maintenance

Other post-transcriptional modifications such as SUMOylation are also shown to contribute to the regulation of CENP-A maintenance. During SUMOylation, the small ubiquitin-like modifier (SUMO) is added to a protein to alter its function (Gareau & Lima 2010; Geiss-Friedlander & Melchior 2007; Mahajan et al. 1997; Matunis et al. 1996; Meluh & Koshland 1995). Similarly to ubiquitination, it may lead to protein degradation. In budding yeast, the aforementioned SUMO-targeted ubiquitin ligase Slx5 is recruited by poly-SUMOylation at the N-terminal domain of Cse4, resulting in Cse4 degradation (Ohkuni et al. 2018).

However, the attachment of a SUMO molecule to a protein can also affect its protein–protein interaction without leading to proteolysis. In humans, SENP6, a SUMO-specific protease that dissociates SUMO molecules from proteins and targets CENP-B and CENP-C, has been shown to regulate the function of kinetochore proteins, as reviewed in (Mitra et al. 2020; van den Berg & Jansen 2023). Upon SENP6 depletion, both kinetochore proteins mislocalised, resulting in decreased CENP-A stability on centromeres (Liebelt et al. 2019; Mitra et al. 2020; Mukhopadhyay & Dasso 2007; van den Berg & Jansen 2023).

Several modifications of CENP-A and its maintenance machinery (not all discussed here) have been revealed so far, with likely more to be reported in the future. The details on their mechanism and significance, as well as the tight interplay between them, are still unknown. However, the loss of those modifications seems to largely decrease genome stability.

## Concluding remarks

The discovery of CENP-A 40 years ago by Bill Earnshaw and colleagues fundamentally transformed our understanding of centromere identity, laying the groundwork for our current understanding of how this specialised chromatin region is defined, organised and maintained.

While as a field we have come a long way towards addressing these questions, some of the molecular pathways underlying these processes still remain as ‘implicated’ rather than ‘fully understood’, and more mechanistic studies using separation-of-function mutants in vitro and in vivo are required to precisely dissect the proposed contributions and roles of various molecular players and associated pathways in maintaining centromere identity. For example, i) how do centromeric transcripts interact with components of the CENP-A loading machinery and how do these interactions translate into the regulation of CENP-A deposition?; ii) what is the molecular basis for the interplay between DNA damage response factors and transcription in maintaining centromere identity?; iii) what are the proteasomal degradation independent roles of ubiquitination and SUMOylation?; and iv) what are the mechanisms defining and protecting the centromere boundary? are some of the timely questions requiring detailed mechanistic understanding. With the ever-growing body of work from the centromere biology community since the discovery of CENP-A, with complementary approaches (from omics to cellular, molecular and mechanistic) and ideas, involving different model organisms, it is highly likely that we will have comprehensive answers to several of these outstanding questions in the near future.

## Data Availability

No datasets were generated or analysed during the current study.
